# Gene Co-expression Network and Regression Analysis Identify the Transcriptomic, Physiological, and Biochemical Indicators of the Response of Alpine Woody Plant *Rhododendron rex* to Drought Stress

**DOI:** 10.3389/fpls.2022.896691

**Published:** 2022-05-25

**Authors:** Xiong-Li Zhou, Jin-Yan Ma, Zhen-Dian Liu, Ni-fei Dai, Hui-Qin Yang, Liu Yang, Yue-Hua Wang, Shi-Kang Shen

**Affiliations:** ^1^School of Ecology and Environmental Science, Yunnan University, Kunming, China; ^2^Yunnan Key Laboratory of Plant Reproductive Adaptation and Evolutionary Ecology, Yunnan University, Kunming, China; ^3^Yunnan Key Laboratory for Plateau Mountain Ecology and Restoration of Degraded Environments, Yunnan University, Kunming, China

**Keywords:** drought response, pectin biosynthesis, woody plant, *Rhododendron*, climate change

## Abstract

Increasing severity of drought stress due to global change and extreme weather has been affecting the biodiversity, function, and stability of forest ecosystems. However, despite being an important component in the alpine and subalpine vegetation in forest ecosystems, *Rhododendron* species have been paid rare attention in the study of molecular mechanism of tolerance or response to drought. Herein, we investigated the correlation of transcriptomic changes with the physiological and biochemical indicators of *Rhododendron rex* under drought stress by using the co-expression network approach and regression analysis. Compared with the control treatment, the number of significantly differentially expressed unigenes (DEGs) increased with the degree of drought stress. The DEGs were mainly enriched in the cell wall metabolic process, signaling pathways, sugar metabolism, and nitrogen metabolism. Coupled analysis of the transcriptome, physiological, and biochemical parameters indicated that the metabolic pathways were highly correlated with the physiological and biochemical indicators under drought stress, especially the chlorophyll fluorescence parameters, such as the actual photosynthetic efficiency of photosystem II, electron transport rate, photochemical quenching coefficient, and the maximum quantum efficiency of photosystem II photochemistry. The majority of the response genes related to the metabolic pathways, including photosynthesis, sugar metabolism, and phytohormone signal pathway, were highly expressed under drought stress. In addition, genes associated with cell wall, pectin, and galacturonan metabolism also played crucial roles in the response of *R. rex* to drought stress. The results provided novel insight into the molecular response of the alpine woody species under drought stress and may improve the understanding of the response of forest ecosystems to the global climate change.

## Introduction

Climate change and extreme weather events have led to rising temperature, and varying precipitation has been widely reported to increase the frequency, magnitude, and spatial extent of drought worldwide (O’Brien et al., 2015; Stott, 2016; Hui-Mean et al., 2018; McDowell et al., 2018; Ahmed et al., 2020). The escalating drought stress is not only a critical threat to the global food production and sustainable agriculture (Zhou et al., 2017; Asseng et al., 2019; Khan et al., 2020), but it also seriously affects the sustainability and stability of ecosystems. For example, massive tree dieback in the forests had happened in Western and Central Europe due to the hottest and driest summer in the region (Orth et al., 2016; Bhusal et al., 2021). Similar drought events have occurred in the United States and Central Europe from 1962 to 1965 (Peterson et al., 2013) and 2018 to 2019 (Brun et al., 2020), respectively. Drought with increasing severity has resulted in the mortality of forests worldwide, especially for the water-sensitive species, and reduced forest productivity. Such phenomenon has changed the structure, distribution, biodiversity, function, and stability of forest ecosystems (Wang et al., 2018; Peltier and Ogle, 2019; He et al., 2020). Therefore, elucidating the mechanism of the tolerance or response of woody tree species to drought would improve the knowledge of the response of forest ecosystems to global climate change.

The response of forest ecosystems to climate changes will partially depend on the ability of forest trees to adapt to the altered environment (Corlett, 2016; Bittencourt et al., 2020). The plasticity of traits of trees to respond to various adverse environments, particularly to drought, has been paid attention. After measuring the plant hydraulic traits, Bittencourt et al. (2020) showed that the Amazonia trees have limited capacity to respond to drought. Photosynthesis and its downstream metabolism compounds, which are susceptible to exotic stress, are the main energy sources for growth and development. De Roo et al. (2020) reported that *Populus tremula* delays photosynthesis and maintains starch reserves under drought stress. Tong et al. (2020) found that the amino acid and sugar metabolisms are the potential metabolism pathways of the response of moso bamboo to drought stress. Zhao et al. (2020) found that the photosystem II photochemistry (Fv/Fm) and photosynthetic efficiency of photosystem II (Y[II]) were decreased in *Paeonia ostii* under drought stress. The stomal conductance and Fv/Fm were also significantly decreased in *Brassica rapa* under drought stress (Greenham et al., 2017). Therefore, elucidating how the gene expression regulates photosynthesis is essential to understand the mechanism of a species’ response to drought stress. In addition, the accumulation of secondary metabolites reflects the response of plants to adverse environments. The flavonoid is an important secondary metabolite for plants to adapt to environments and for self-protection (Tong et al., 2020). Previous studies have demonstrated that genes involved in the biosynthesis of flavonoids was significantly enriched under drought stress, suggesting that flavonoids may enhance the species’ tolerance of drought (Tong et al., 2020; Xu et al., 2021). Malondialdehyde (MDA), proline, and total soluble sugar characterize the oxidative damage of plants when subjected to exotic stress (Wu et al., 2017; Rathore et al., 2018; Tong et al., 2020). Previous studies have found that species could regulate and change the content of these compounds, which act as osmoprotectant response to drought stress (Cai et al., 2019; Tong et al., 2020). During plant growth and development, the cell wall plays a vital role in the perception and regulation of exotic signals (Singh et al., 2017). Pectin is a critical component in the cell wall and could change the structure and function of the cell wall. Fox et al. (2018) reported that after water recovery, the cell wall related to transcripts (expansin) was enriched in *Pinus halepensis* under drought stress. Thus, the response of plants to drought stress always involved in multi-level’s mechanism.

*Rhododendron* L. is a genus of perennial woody plants in the Ericaceae family and consists of approximately 1,025 species. These species are distributed across the northern temperate zone, throughout tropical Southeast Asia, and northeastern Australia (Chamberlain et al., 1996; Zhou et al., 2020). *Rhododendron* species are not only famous woody ornamental plants worldwide but are also indispensable components in the alpine and subalpine vegetation in China (Zhang et al., 2017). This genus has irreplaceable economic and ecological values in the biodiversity and the stability and sustainability of an ecosystem, especially for forest ecosystems (Zhang et al., 2017; Shrestha et al., 2018). Although previous studies demonstrated that the growth and development of *Rhododendron* seedling is susceptible to soil water, it has received rare attention in the response to drought stress under global changes. Herein, the transcriptomic, physiological and biochemical indicators of *Rhododendron rex* were evaluated by weighted gene co-expression network analysis and regression analysis to determine the molecular mechanism of the response to drought stress. This study was performed to determine the physiological and biochemical indicators and metabolic pathways of the response of *R. rex* to drought stress. The mechanism by which these indicators were mediated by the regulation of gene expression involved in the metabolic pathways in the response to drought stress was also elucidated. The results will provide novel insights into the understanding of the physiological and biochemical indicators, changes in gene expression, and metabolic pathways in the response of *Rhododendron* to drought stress.

## Materials and Methods

### Plant Materials and Treatments

*Rhododendron rex* seedlings with similar size and growth periods were collected from Laojunshan (altitude: 3,500 m; location: 26°39′00.1″N; 99°46′27.8″E) in Yulong County, Yunnan Province in June 2020. The collected seedlings were planted in culture pots (19 × 27 × 23 cm^3^) with ~5.0 kg of soil per pot, cultivated and domesticated with daily normal watering for 2 months in the greenhouse (temperature: 15–25°C; humidity: 55–60%) of Yunnan University, Yunnan Province, China. Then, the drought treatments were conducted according to Du et al. (2020), Jia et al. (2020), and Zhang et al. (2021). Specifically, the soil moisture of the field water-holding capacity was kept at 75–80% (control, CK), 55–60% (light drought stress, D1), 40–45% (moderate drought stress, D2), and 30–35% (severe drought stress, D3). In different drought stress, the pots were weighed every day and re-watered to sustained field water-holding capacity (Yang et al., 2010; Al-Yasi et al., 2020). Three replicates with five seedlings in each replicate were performed. Due to our previous finding that seedling of *R. rex* was extremely sensitive to water stress, the samples were taken after 3 days of drought treatments (Unpublished data). The leaves were sampled, cleaned, and immediately placed in liquid nitrogen for transcriptomic sequencing.

### Measurement of Chlorophyll Fluorescence Parameters

Mini-PAM (Walz, Effeltrich, Germany) was used to determine the photosynthetic characteristics. In accordance to Liu et al. (2019), the following chlorophyll fluorescence parameters were determined: minimal fluorescence from dark-adapted leaf (*F*_0_), maximum fluorescence from dark-adapted leaf (Fm), Fv/Fm, Y(II), photochemical quenching coefficient (qP), electron transport rate (ETR), and nonphotochemical quenching (NPQ). The photosynthetic characteristics and chlorophyll fluorescence parameters were determined using nine different leaves (three mature leaves were selected from each individual) for each treatment.

### Measurement of Physiological and Biochemical Indicators

The nitrogen balance index (NBI), flavoroids (Flv, μg/cm^2^), and anthocyanin (Anth, μg/cm^2^) were measured by the Dualex scientific^+^. The chlorophyll contents of were assayed according to the method described by Lichtenthaler and Wellburn (1983). The leaf (~0.1 g) was cut into pieces, added with 10 ml of 80% acetone, and soaked for 48 h at room temperature in the dark. Then, the supernatant was determined at 663 and 645 nm to evaluate the chlorophyll, with 80% acetone as the blank control. Chlorophyll *a* and chlorophyll *b* were calculated as follows: Chla (mg/L) = 12.72 × A663–2.59 × A645; and Chlb (mg/L) = 22.88 × A645–4.67 × A663. The total soluble sugar was estimated following the anthrone method (Yemm and Willis, 1954). The MDA content was determined according to Hodges et al. (1999), with some modifications. The leaf tissue (~1 g) was homogenized in 5 ml of 5% (*w*/*v*) TCA and centrifuged at 3,000 rpm for 10 min. Then, 2 ml of the supernatant was collected from the tube, and 2 ml of 0.67% TBA [prepared in 20% TCA (*w*/*v*)] was added. The mixed samples were heated at 95°C for 30 min. After cooling, the absorbance was determined at 450, 532, and 600 nm. The proline content was determined by proline-acid ninhydrin according to Bates et al. (1973), with slight modifications. The leaf tissue (~0.5 g) was homogenized with 5 ml of 3% aqueous sulfo-salicylic acid in boiling water bath for 10 min. Then, 2 ml of the supernatant was added to glass tubes with 2 ml of glacial acetic acid and 2 ml of acid ninhydrin. The mixture was boiled at 95°C for 30 min. After cooling, 4 ml of toluene was added for extraction. The tubes were shaken for 30 s and undisturbed for 30 min at room temperature. The absorbance of the supernatant was determined at 520 nm, with proline as the standard. The absorbance was determined by a UV-vis spectrophotometer (Evolution 220; Thermo Scientific, Waltham, MA, United States). Three replicates of each physiological and biochemical indicator were determined in each drought treatment.

One-way ANOVA was performed to compare the physiological and biochemical indicators between the drought treatments. If the normality test and homogeneity of variance passed, ordinary one-way ANOVA was performed. Otherwise, Kruskal–Wallis test was performed. Differences between the values were considered significant at *p* < 0.05.

### RNA Sequencing, Library Construction, Sequence Assembly, and Annotation

Four leaves from four seedlings in each drought treatment representing four biological replicates were sampled, frozen in liquid nitrogen, and stored at −80°C until RNA extraction. A total of 16 samples were collected for RNA extraction. Total RNA was extracted using a TRIzol reagent (Invitrogen, CA, United States) according to the manufacturer’s instructions. An RNA-seq library was constructed by processing at least 1 μg of RNA per sample into cDNA libraries by using a Truseq RNA sample preparation kit (Illumina, CA, United States). Then, the libraries were sequenced in an Illumina HiSeq4000 sequencing platform to produce two 150-bp paired-end reads. The quality of the RNA-Seq data was validated by performing RT-qPCR analysis on an ABI7500 fluorescent quantitative PCR machine (Applied Biosystems, United States). The relative expression of each gene was normalized through the 2^−ΔΔ*C*t^ method. The mRNA abundances of the targeted genes were normalized to the CYP gene (Yi et al., 2012). All primers for the qRT-PCR are listed in Supplementary Table S1.

The raw reads were filtered by discarding the contaminated adaptors by using the default steps in SeqPrep.^1^ The default parameters of Trinity^2^ were used to assemble the clean reads. To determine the duplication levels of the clean reads, the Q20, Q30, and guanine cytosine (GC) contents were calculated for the sequences (Grabherr et al., 2011), and the transcripts per million reads (TPM) mapped reads were generated by the software package of RSEM (Li and Dewey, 2011). The gene annotation was analyzed using publicly available protein databases with nonredundant databases, Swiss-Prot database, and evolutionary genealogy of genes, including the Nonsupervised Orthologous Groups, Gene Ontology (GO), Pfam, and Kyoto Encyclopedia of Genes and Genomes (KEGG) by using DIAMOND (Buchfink et al., 2015), BLAST2GO (Conesa et al., 2005), HMMER (Finn et al., 2011), and KOBAS2.1 (Xie et al., 2011), respectively (Supplementary Table S2). Raw transcriptome sequences data have been deposited at NCBI Sequence Read Archive (SRA) under BioProject PRJNA738298.

### Identification and Enrichment Analysis of the Differentially Expressed Unigenes

To identify the differentially expressed unigenes (DEGS) by using DESeq2 (Love et al., 2014), the parameter was set to *p*-adjust of <0.05 and |log_2_FC| ≥ 2, and the *p* values were corrected using the method of Benjamini and Hochberg. To determine the biological functions of the unigene sets, Fisher’s exact test was performed for the GO and KEGG enrichment analysis, and the *p* value corrected with false discovery rate was <0.05. The data were analyzed on the free online platform of Majorbio Cloud Platform.^3^

### Co-expression Network Analysis and Correlation of Physiological and Biochemical Indicators

To determine the specific expressed unigene sets associated with traits, the weighted gene co-expression network analysis (WGCNA) was performed. To preprocess and filter the data, the parameter was set to TPM < 2, and the threshold of the variation coefficient of expression in the *R. rex* was <0.1. The soft threshold of 6 was selected based on the scale-free topology. Then, the adjacency matrix was converted to the topological overlap matrix. Based on the similarity algorithm, the unigenes were clustered, and the parameter of mergeCutHeight 0.2 was set to define the modules. For each module, the module was evaluated using principal component analysis (PCA), and the first principal component (PC1 is the eigengene for each module) was analyzed by linear regression (GraphPad Prism trial version 9.0) to determine the modules associated with the physiological and biochemical indicators. In addition, the correlation of each module and traits were calculated using the Spearman correlation coefficient. The modules were selected (*p* < 0.05) to perform the GO and KEGG enrichment analysis. The heatmap was visualized by TBtools (Chen et al., 2020).

## Results

### Effect of Drought Stress on the Physiological and Biochemical Indicators

**Figure 1:**
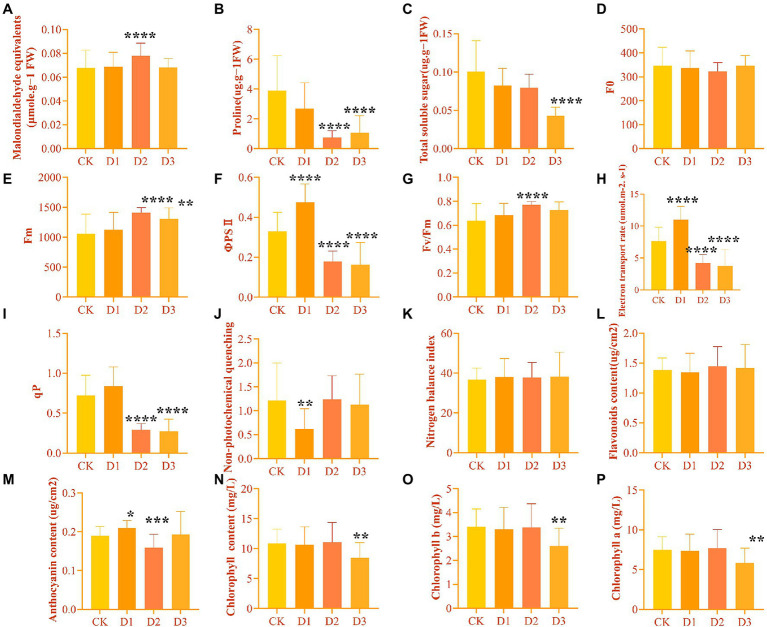
The physiological and biochemical indicators differences under drought treatments were observed by one-way ANOVA analysis in *Rhododendron rex*. **(A)** malondialdehyde equivalents (MDA, μmol⋅g^−1^ FW); **(B)** proline (Pro, μg⋅g^−1^ FW); **(C)** total soluble sugar (TSS, μg⋅g^−1^ FW); **(D)** the minimal fluorescence from dark-adapted leaf (*F*_0_); **(E)** the maximum fluorescence from dark-adapted leaf (Fm); **(F)** the maximum quantum efficiency of photosystemIIphotochemistry (Fv/Fm); **(G)** electron transport rate (ETR, μmol⋅m^−2^⋅s^−1^); **(H)** photochemical quenching coefficient (qP); **(I)** nonphotochemical quenching (NPQ); **(J)** nitrogen balance index (NBI); **(K)** flavoroids (Flv, μg/cm^2^); **(L)** anthocyanin (Anth, μg/cm^2^); **(M)** chlorophyll (Chl, mg/L); **(N)** chlorophyll *b* (Chl *b*, mg/L); **(O)** chlorophyll *a* (Chl *a*, mg/L). The figures were analyzed and visualized by GraphPad Prism (trial version 9.0). The *p* values were showed by symbol (^*^p < 0.05; ^**^p < 0.01; ^***^p < 0.001; ^****^p < 0.0001).

The leaf physiological and biochemical indicators of *R. rex* showed different changes in the response to drought stress. As expected, the Y(II), ETR, and qP under D2 and D3 treatments were significantly decreased. However, these chlorophyll fluorescence parameters were higher in D1 treatment than in CK (Figure 1). Similar variations in Fm and Fv/Fm were found among the different treatments, and the significant highest values were found in the D2 treatments (Figure 1). However, NPQ was significantly lower in the D1 treatments than in the other treatments. Furthermore, the content of chlorophyll and TSS decreased with the intensifying degree of drought stress, with the significantly lowest values in the D3. Meanwhile, MDA was significantly higher in D3 treatment than in the other treatments, while proline was significantly lower in D3 than in the CK and D1 treatments (Figure 1). No significant differences in some parameters, such as the Y(II), ETR, and anthocyanin content, were found between the D1 treatment and CK. This result indicated that *R. rex* may have a certain ability to resist drought.

### RNA-Seq Data Analysis

An average of 49 million clear reads was obtained from the constructed cDNA libraries after quality check and data filtering, with averages of 97.40 and 92.86% for Q20 and Q30 bases, respectively. The average GC content was 46.75% (Supplementary Table S2). Assembly for unigenes and transcripts was performed using the *de novo* assembly tool Trinity. An average of 255,300 transcripts was identified with the average length of 1,034.85 bp and the N50 length of 1,754 bp. All the transcripts from the constructed library were assembled to obtain the average of 148,136 unigenes with the average length of 918.42 bp and the N50 length of 1,525 bp. Seven genes from each drought stress treatment were selected for RT-qPCR analysis to validate the quality of the RNA-seq data (Supplementary Figure S1). The similar expression trends of the RNA-seq data, except in two genes (*psbS*, *HSP90A*), and RT-qPCR data indicated the reliability of the present transcriptomic profiling data (Supplementary Figure S1).

### Identification of DEGs Under Different Drought Stress

We identified the DEGs based on the pairwise comparison between CK and different drought treatments. Consequently, 176 (77 up-regulated and 99 down-regulated), 228 (114 up-regulated and 114 down-regulated), and 1,132 (515 up-regulated and 617 down-regulated) significant DEGs were detected in the D1, D2, and D3 treatments when compared with CK, respectively. The number of DEGs increased with the intensifying degree of drought treatments.

### Function Annotation of the DEGs Under Different Drought Stress

**Figure 2:**
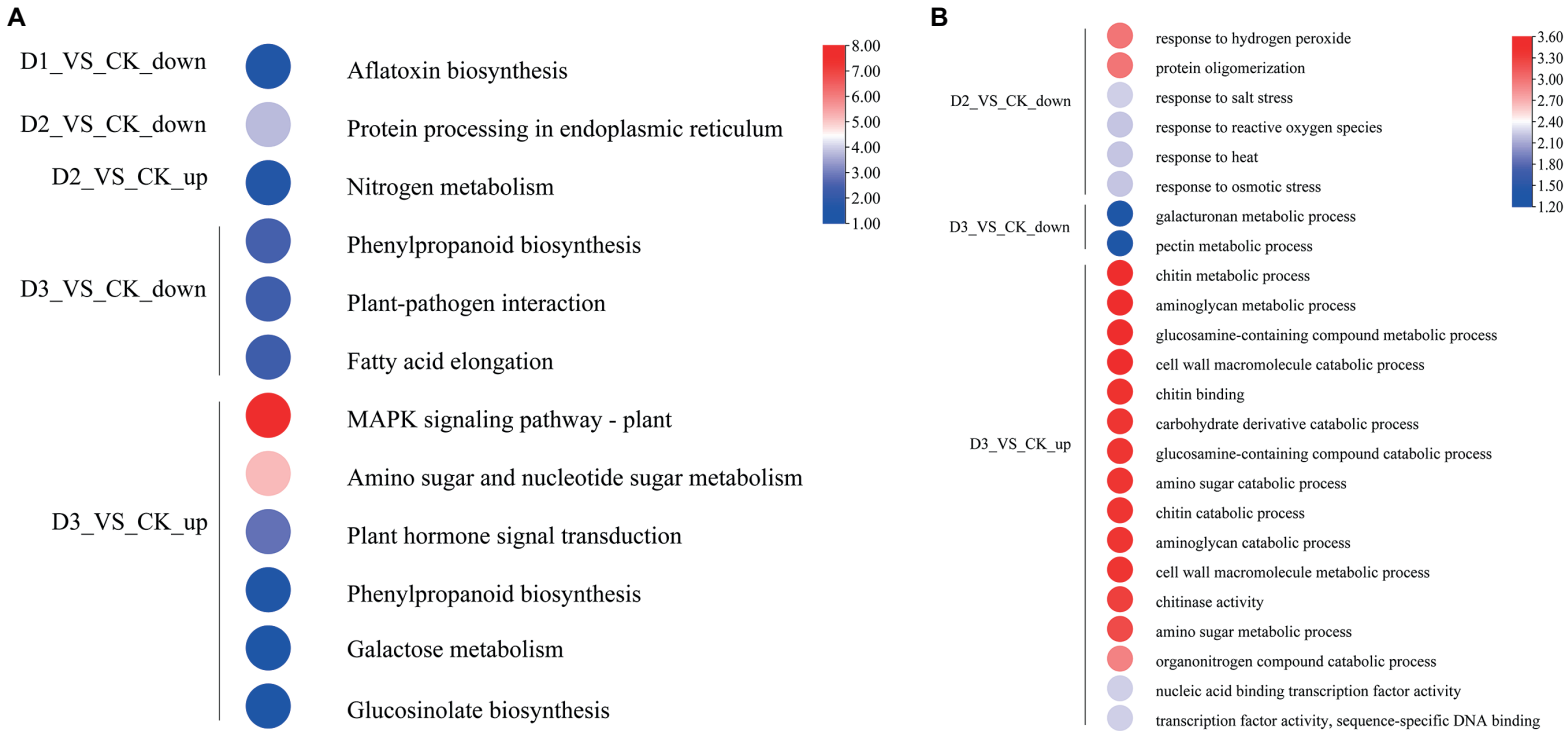
The significant differentially expressed unigenes was significant enrichened (*p* value corrected <0.05) of GO and KEGG pathways. **(A)** KEGG enrichment pathways; **(B)** GO enrichment items. The color was represented −log (*p* value corrected), the red was showed the low *p* value corrected and the blue was high *p* value corrected. The up is up-regulated unigenes and the down is down-regulated unigenes. The figures were visualized by TBtools.

**Figure 3:**
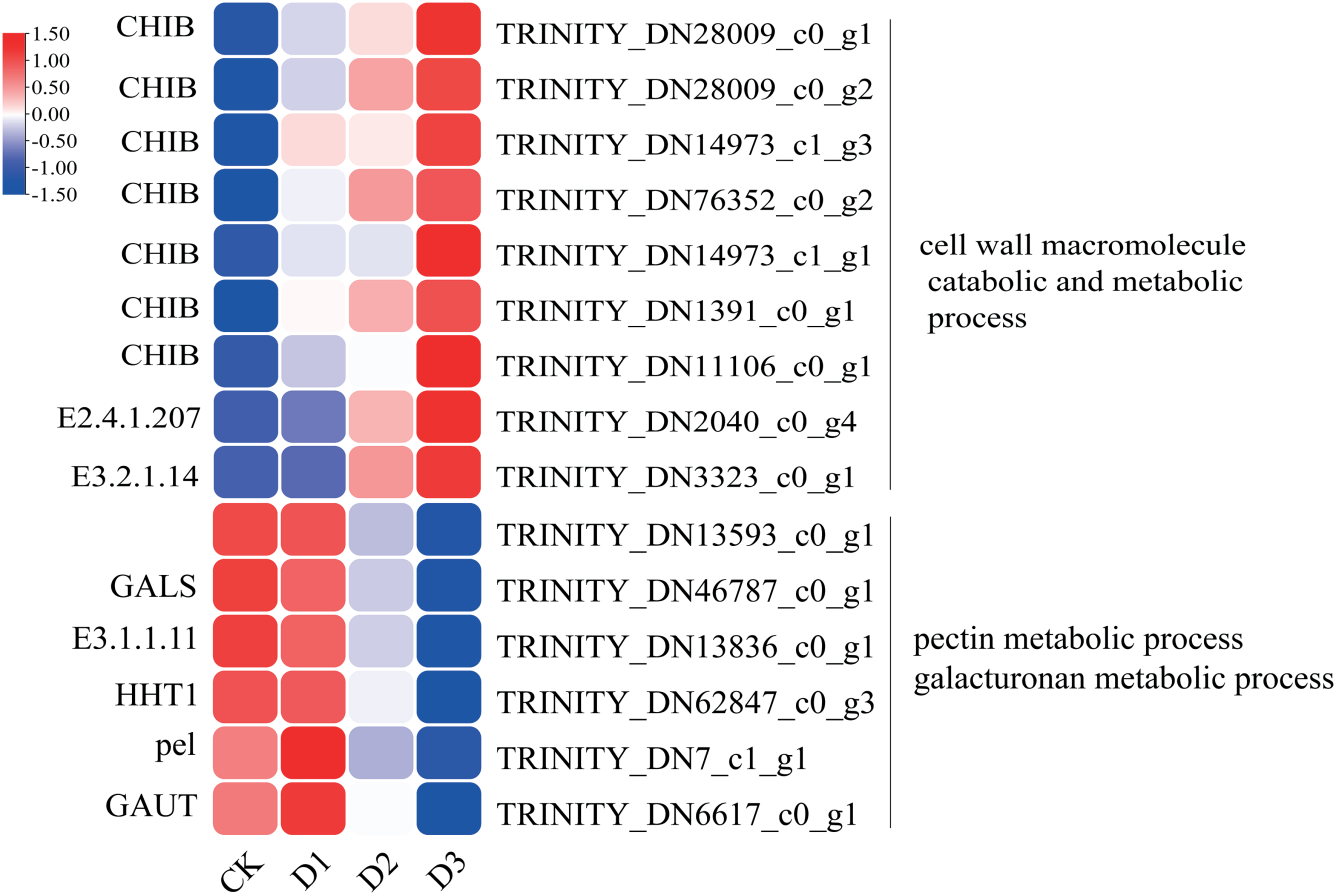
The significant differentially expressed unigenes related to cell wall and pectin was significant enriched of GO items in the CK verse D3. The color was showed the expression values (log_10_(TPM)), and the red represent high TPM. The figures were visualized by TBtools.

The detailed biological functions of these DEGs were analyzed by GO and KEGG pathway enrichment analysis. The results showed that the pectin and galacturonan metabolism and cell wall macromolecular compound metabolic and catabolic processes were enrichened in the GO items (Figure 2B; Supplementary Figure S2). Thus, the genes associated with the cell wall metabolic process may have played an important role in the response of *R. rex* to drought stress. Further analysis showed that the expression levels of genes (i.e., *CHIB*, *E2.4.1.207*, and *E3.2.1.14*) involved in the cell wall macromolecular compound metabolic and catabolic process were significantly upregulated in the D3 treatments. Conversely, the genes (i.e., *GALS*, *E3.1.1.11*, *HHT1*, *pel*, and *GAUT*) involved in pectin and galacturonan metabolic process were significantly downregulated in the D3 treatments (Figure 3). However, similar expression levels of these genes were detected between CK and D1 treatments (Figure 3), indicating that the genes expression levels were slightly affected by D1 treatments. These results supported that *R. rex* had a certain tolerance to water deficit.

**Figure 4:**
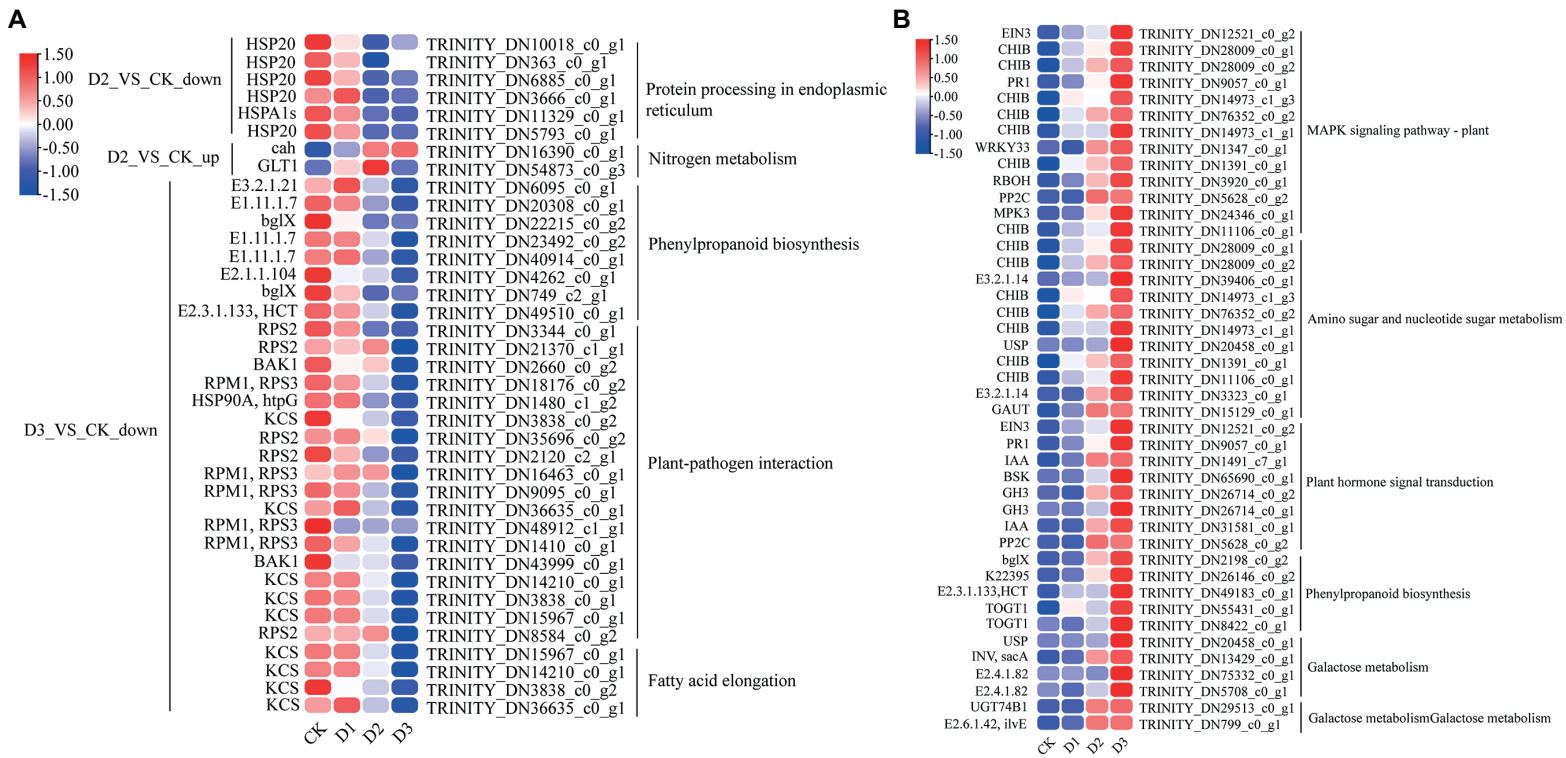
The significant differentially expressed unigenes was significant enrichened of KEGG pathways. The **(A)** is the up- and down-regulated differentially expressed unigenes of D2 and D3 verse CK. The **(B)** is the up-regulated differentially expressed unigenes of D3 verse CK. The color was showed the expression values (log_10_(TPM)), and the red represent high TPM. The figures were visualized by TBtools.

KEGG enrichment analysis showed that the DEGs involved in nitrogen metabolism, phenylpropanoid biosynthesis, fatty acid elongation, MAPK signaling pathway, plant hormone signal transduction, galactose metabolism, and amino sugar and nucleotide sugar metabolism were significantly enriched (Figure 2A). The expression levels of genes (i.e., *HSP20, E3.2.1.21, HSP90A, RPS2*, and *KCS*) were significantly downregulated in the D3 treatments (Figure 4A), while the genes (i.e., *CHIB, cah, MPK3, PP2C, WRKY33, USP, E3.2.1.14, GAUT, IAA, BSK, GH3*, and *INV*) involved in the MAPK signaling pathway, plant hormone signal transduction, galactose metabolism, nitrogen metabolic, phenylpropanoid biosynthesis, and amino sugar and nucleotide sugar metabolism were significantly upregulated in the D3 treatments (Figure 4B). These results suggested that the signaling pathways, sugar metabolism, nitrogen metabolism, phenylpropanoid biosynthesis, and fatty acid elongation played important roles in the response of *R. rex* to drought stress.

### Gene Co-expression Network Construction and Regression Analysis

**Figure 5:**
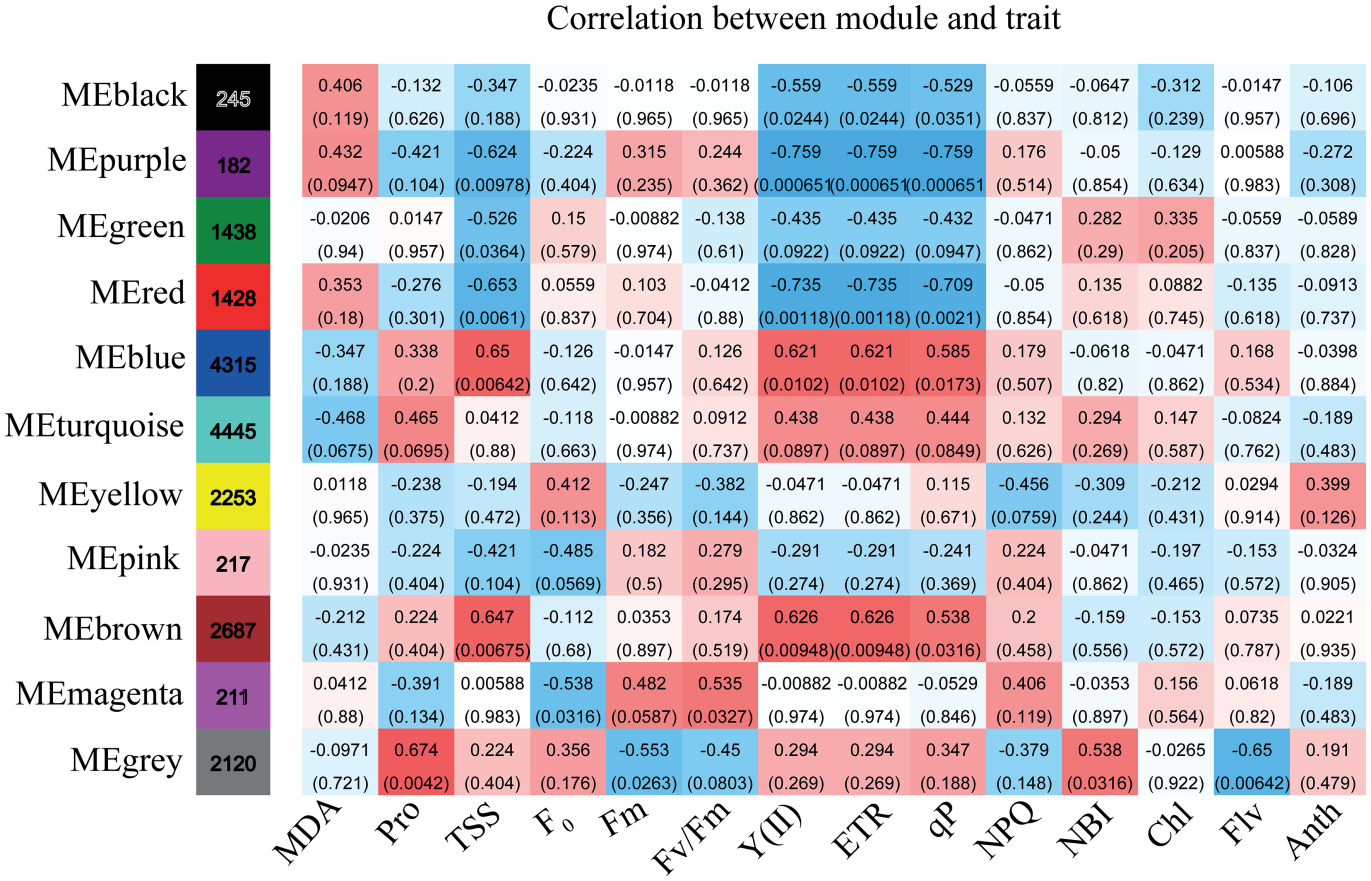
The changes in physiology under drought stress are correlated with co-expression networks. Each row corresponds to a module labeled with a color, and it is distinguished by different colors which were arbitrarily assigned by the WGCNA package. Each column corresponds to a tissue type as indicated. The color of each cell at the row–colum intersection indicates the correlation coefficient (R) between the module and the tissue type.

WGCNA was applied to relate the genes with the physiological and biochemical indicators under the different drought treatments. Eleven classification modules were acquired based on the expression patterns of genes during drought treatments (Supplementary Figure S3). The correlation of the module eigengenes with the physiological and biochemical indicators were calculated by using Spearman. Eight modules (i.e., black, purple, green, red, blue, brown, magenta, and gray) had significant positive or negative correlation with the various physiological and biochemical indicators (Figure 5). The black, purple, and green modules were negatively associated with Y(II), ETR, and qP, and the purple and green modules were negatively correlated with TSS. The purple module also had negative correlation with chlorophyll. Conversely, the blue and brown modules had significant positive correlation with Y(II), ETR, qP, and TSS, while the gray module had significant positive correlation with chlorophyll and proline.

**Figure 6:**
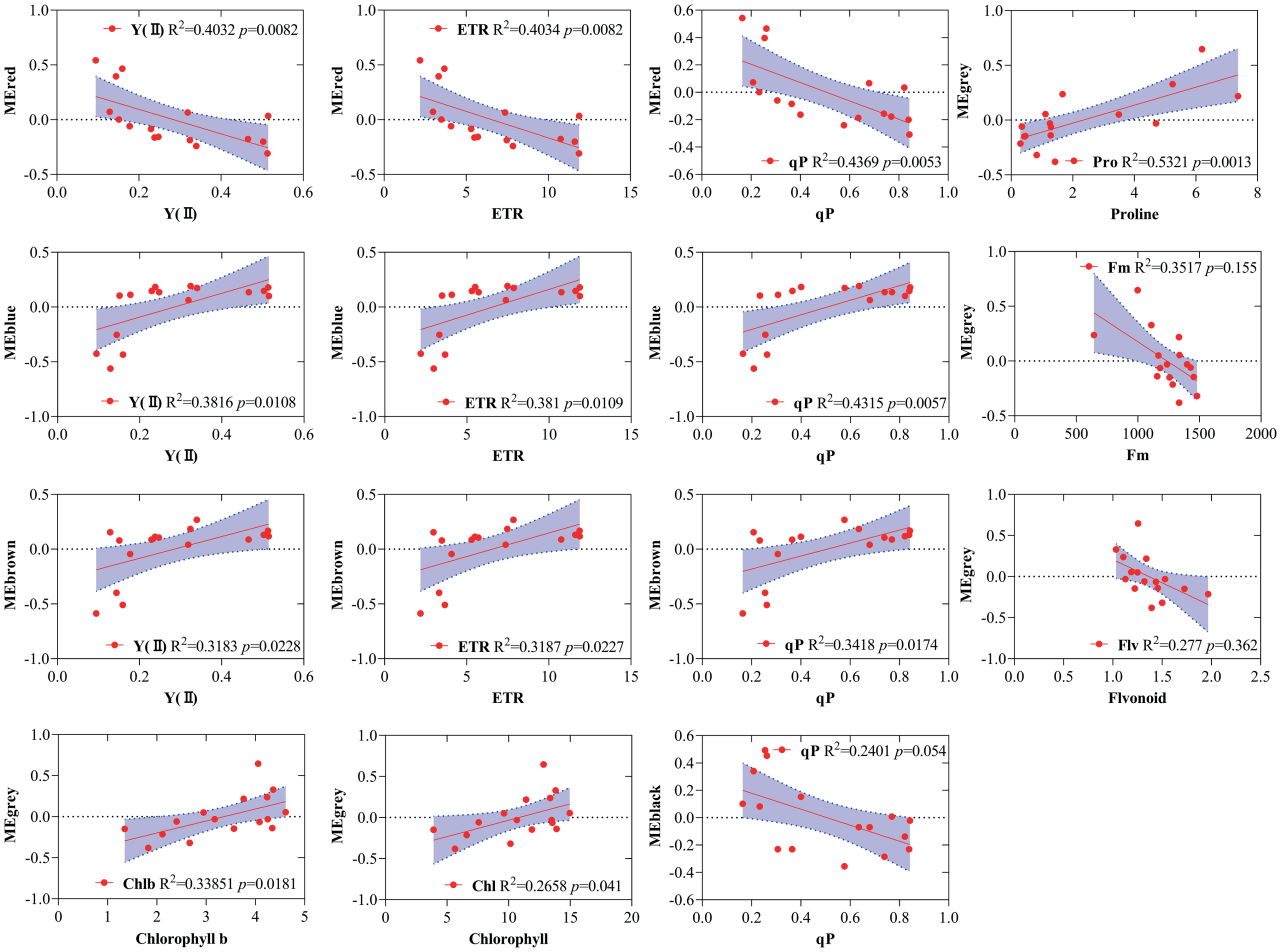
Correlations between specific gene module eigen gene values (first principle component as a representative of the entire module) and corresponding physiological and biochemical indicators for MEred, MEblue, MEbrown, MEgrey. The red line indicates the regression line and lavender shading indicates the 95% confidence interval.

Simple linear regression analysis was performed to further calculate the correlation between the modules and the physiological and biochemical indicators. The result showed that the red, blue, and brown modules were significantly correlated with Y(II), ETR, and qP, and the black module was significantly correlated with qP (Figure 6). Five traits, namely, proline, Fm, flavonoid, chl *b*, and chlorophyll, were correlated with the gray module. The results reveal that the genes of several modules were involved in regulating the physiological and biochemical processes of the response of *R. rex* to drought stress.

### Correlating Modules With the Physiological and Biochemical Indicators

**Figure 7:**
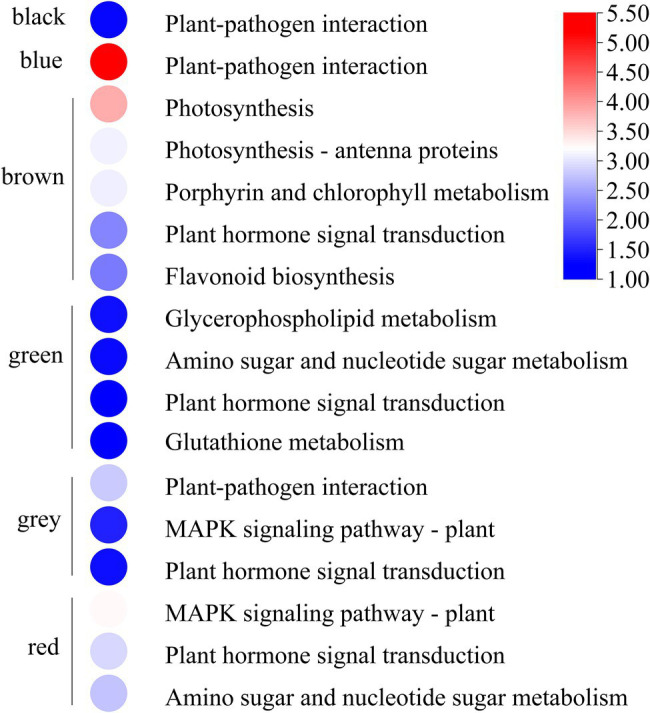
The modules were significant enriched (*p* value corrected <0.05) KEGG pathways. The color was represented −log (*p* value corrected), the red was showed the low *p* value corrected and the blue was high *p* value corrected. The figures were visualized by TBtools.

**Figure 8:**
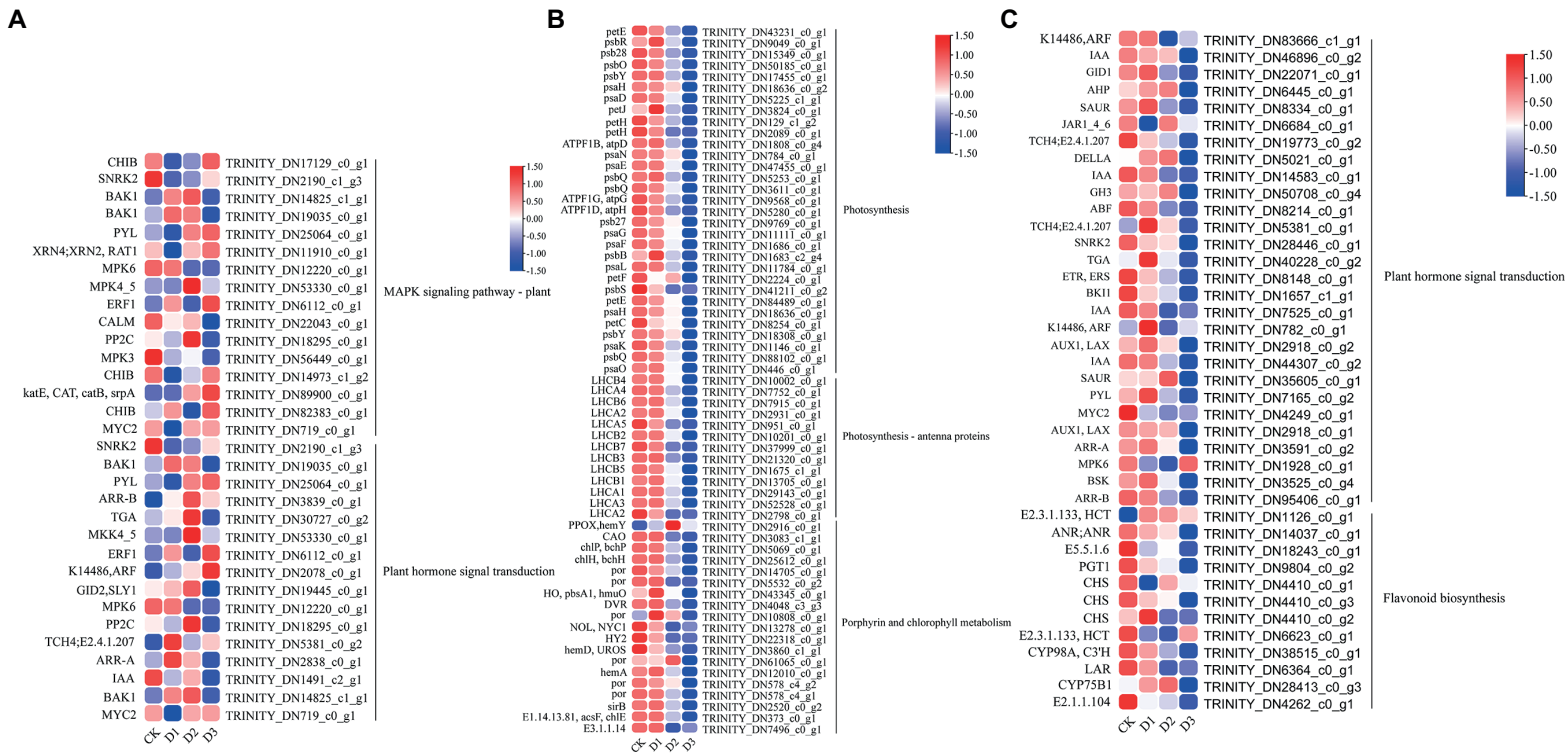
The module unigenes correlation with physiological and biochemical indicators was significant enriched of KEGG pathways. **(A)** is the grey module, **(B,C)** is the brown module. The color was showed the expression values (log_10_(TPM)), and the red represent high TPM. The figures were visualized by TBtools.

**Figure 9:**
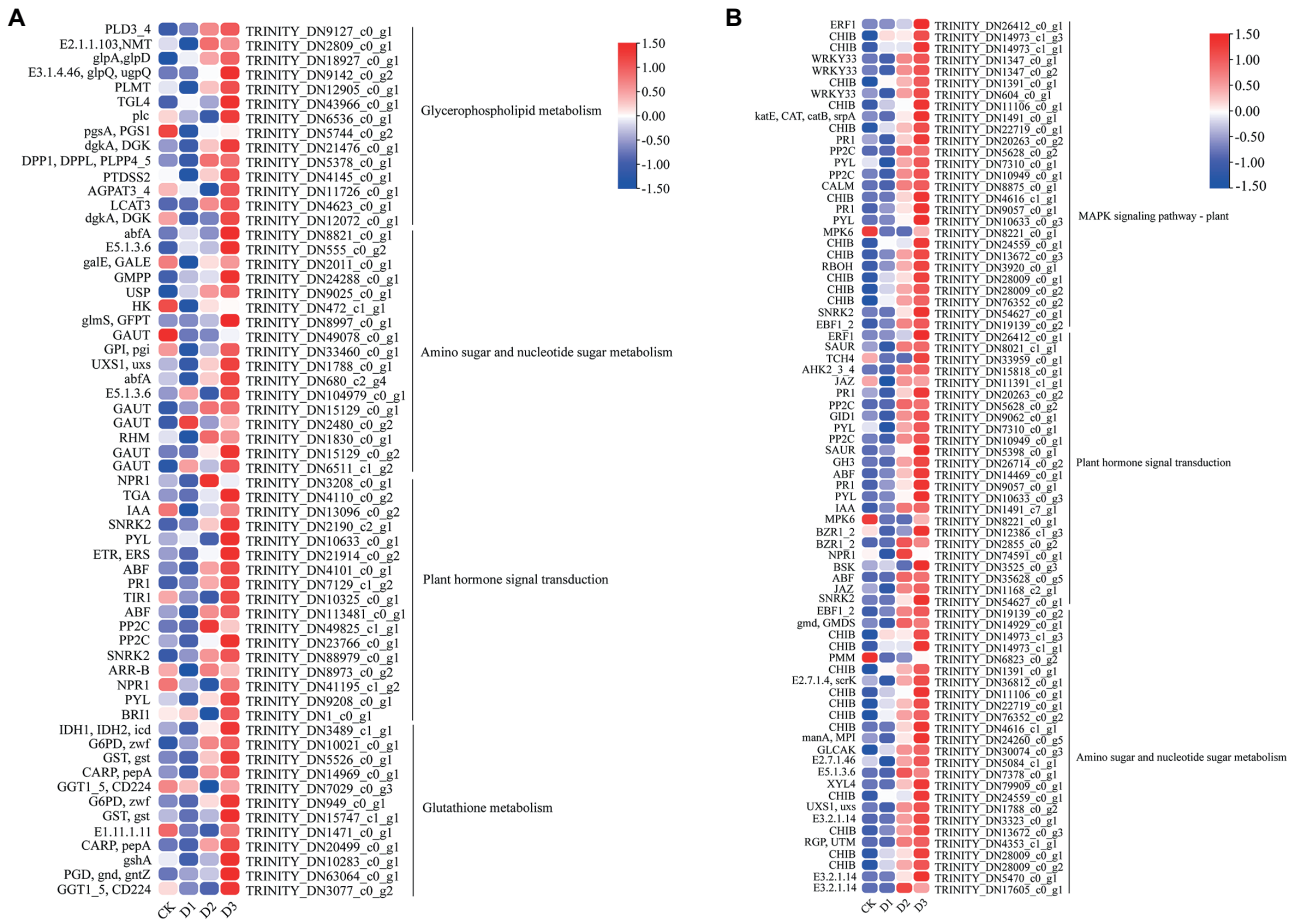
The module unigenes correlation with physiological and biochemical indicators was significant enrichened of KEGG pathways. The **(A)** is the green module, **(B)** is the red module. The color was showed the expression values (log_10_(TPM)), and the red represent high TPM. The figures were visualized by TBtools.

GO and KEGG enrichment analyses were performed to determine the biological process of the genes in different modules related to the physiological and biochemical indicators. The results showed that the genes in the brown module were significantly enriched in the photosynthesis process, biosynthesis of pectin, antenna proteins, porphyrin and chlorophyll metabolism, plant hormone signal transduction, and biosynthesis of flavonoids (Supplementary Figures S4, S5B and Figure 7). Meanwhile, the expression levels of the genes, such as *por*, *psaD, psb28, petE*, and *LHCB2*, involved in photosynthesis, signaling pathways, and biosynthesis of flavonoids showed a decreasing trend with the intensifying degree of drought treatments (Figure 8). Regression analysis showed that the gene expression patterns in the brown module had significant correlations with Y(II), ETR, and qP (Figure 6). Genes in the red module were significantly enriched for the MAPK signaling pathway, plant hormone signal transduction, and amino sugar and nucleotide sugar metabolism (Figure 7). However, the genes involved in these pathways exhibited increasing expression levels in the D2 and D3 treatments of *R. rex* (Figure 9). The red module also showed a trend suggesting its correlation with Y(II), ETR, and qP (Figure 6). Two modules (black and blue) were significantly enriched in the plant–pathogen interaction, and the gene expression patterns in the black and blue modules had significant negative and positive correlation with qP, respectively (Figures 6, 7). In addition, significant positive correlations were found between the blue module and Y(II) and ETR. The expression levels of genes involved in the plant–pathogen interaction in the black module were increased with the intensifying degree of drought treatments (Figure 8). Conversely, these genes showed low expression levels in the D2 and D3 treatments in the blue module. Genes in the green module were significantly enriched in the amino sugar and nucleotide sugar metabolism, glutathione metabolism, plant hormone signal transduction, and glycerophospholipid metabolism (Figure 7). However, a significant correlation was not observed between the physiological and biochemical traits and the expression trend of genes in the green module. The gray module was enriched in the MAPK signaling pathway, plant hormone signal transduction, and plant–pathogen interaction. Furthermore, significant positive correlations were found between the expression patterns and proline, chl *b*, and chlorophyll in the gray module (Figure 6). Conversely, the grey module had significant negative correlation with Fm and flavonoid. These results suggested that the expression of genes involved in photosynthesis, signaling pathways, sugar metabolism, and the biosyntheses of flavonoids and pectin were regulated during the response of *R. rex* to drought stress.

## Discussion

Drought stress resulting from the global climate change has recently received extensive attention, especially in crops and model plant. However, as an important component in alpine and subalpine vegetation in forest ecosystems, *Rhododendron* species have received rare attention in the understanding of the mechanism of drought tolerance/response. Here, we reported the molecular response mechanisms of *R. rex* to drought stress through WGCNA and regression analysis. We found that genes associated with photosynthesis, sugar metabolism, and phytohormones signal pathways participate in the response of *R. rex* to drought stress. In addition, we also found that genes associated with the cell wall, pectin, and galacturonan metabolism play crucial roles in the response of *R. rex* to drought stress. The results will provide a novel knowledge on the response of alpine woody species to drought stress resulting from global changes.

Photosynthesis is susceptible to drought stress, resulting in decreased energy and yield (Greenham et al., 2017; Zhao et al., 2020). Previous studies have shown that species could modulate the changes in the chlorophyll fluorescence parameters to optimize its photosynthetic capacity when subjected to drought stress (García-Plazaola et al., 2017; Greenham et al., 2017). Our results showed that Y(II), ETR, and qP were significantly decreased under drought treatments. In general, most plant species with healthy photosynthetic tissues exhibit a mean Fv/Fm of 0.80 (Bjorkman and Demmig, 1987). The value of Fv/Fm always decreased when the plant subject to extotic stress. However, the present study found that the values of Fv/Fm increased under drought stress (Figure 1). Cai et al. (2015) also found that the value of Fv/Fm of *Rhododendron delavayi* increased compared to control after 4 days of water stress. Thus, the chlorophyll fluorescence of *Rhododendron* species response to exotic stress needs further study from the perspective of temporal dynamics.

Genes involved in photosynthesis, antenna proteins, porphyrin, and chlorophyll were down-regulated under drought stress (Figure 9). The light-harvesting chlorophyll protein complex (LHC) is embedded in the membrane of the chlorophyll thylakoid. LHCA (i.e., LHCA1, LHCA2, LHCA3, LHCA4, and LHCA5) is located in the photosystem I (Caspy and Nelson, 2018), which mainly functions for converting light energy to chemical energy. LHCB (i.e., LHCB1, LHCB2, LHCB3, LHCB4, LHCB5, LHCB6, and LHCB7) in the photosystem II can control the light-harvesting process and protect the primary photochemical (Andersson et al., 2003). In addition, the LHC protein complex contains some subunits, including Psb27, Psb28, PsbO, PsbQ, PsbS, and PsbY, in the photosystem II and PsaE, PsaF, PsaL, and PsaK in the photosystem I. These subunits are known to drive the collection and conversion of light energy and protect from photodamage in the photosystem, such as the subunits of Psb27 and Psb28 that protect the photosystem from excess photodamage during the electron transport process (Bečková et al., 2017; Weisz et al., 2017; Zabret et al., 2021). The decrease in chlorophyll content results in reducing light absorption, and this process affects the functional reaction centers of the photosystem (Andersson et al., 2003). In the present study, the chlorophyll, chl *a*, and chl *b* were significantly decreased under the drought stress treatments (Figure 1), indicating that *R. rex* may protect the reaction center from damage by reducing the chlorophyll contents, capture of the light energy, and electron transfer efficiency of photosystems I and II under drought stress. In addition, the genes for photosynthesis electron transport (*petE*), ferredoxin (*petF*)*, petJ*, and ferredoxin–NADP reductase (*petH*) were involved in the electron transport process (Sharon et al., 2009). The decreased expression levels of these genes result in the limited light absorption and transformation in *R. rex* under drought stress. Moreover, regression analysis showed that the expression patterns of these genes had significant positive correlation with Y(II), ETR, and qP. Therefore, the modifications in the expression of the genes involved in photosynthesis would participate in the response of *R. rex* to drought stress by decreasing the ETR, actual photosynthetic efficiency, qP, and chlorophyll content.

Sugar is one of the major energy sources of plants, and the sugar metabolism includes starch and sucrose metabolism, glycolysis or gluconeogenesis, galactose metabolism, and amino sugar and nucleotide sugar metabolism (Tong et al., 2020). Previous studies have proposed that sugar metabolism plays a vital role in the osmotic regulating process under drought stress (Conde et al., 2015; Tong et al., 2020). Genes associated with galactose and amino sugar and nucleotide sugar metabolism were found to be significantly enriched, and the genes (i.e., *USP, INV*, and *E2.4.1.82*) involved in these metabolism processes were upregulated under drought stress in the present study (Figures 4, 8). Therefore, as an osmoprotectant, sugar may accumulate in the seedlings of *R. rex* subjected to drought stress (Rangani et al., 2020; Tong et al., 2020). In addition, the UDP-sugar pyrophosphorylase (*USP*) and other genes, such as *CHIB, gmd, manA, XYL4, RGP, E2.7.1.46, E5.1.3.6*, and *E3.2.1.14*, were also up-regulated in the *R. rex* under different drought treatments (Figure 8). The *E5.1.3.6* gene is involved in the UDP-D-galacturonic acid synthesis (Zhou et al., 2020), and *USP* mediated galactose-1-phosphate synthesis of the UDP-galactose under the galactose metabolism (Damerow et al., 2015). Thus, the upregulation of these genes suggested that the sugar metabolism on the molecular level was regulated during the response to drought treatments.

Flavonoids are not only important secondary metabolites but are also vital substances in the adaptation to environments and self-protection (Tong et al., 2020). Flavonoid accumulation can enhance the ability to resist drought in different ways. First, the flavonoids could serve as antioxidants and scavenge free radicals, especially anthocyanins (Ahmed et al., 2015; Tong et al., 2020). Second, flavonoids participate in the osmotic regulating process to respond to drought stress (Zhao et al., 2017). Finally, flavonoids are also beneficial for the light reaction during photosynthesis to reduce the influences of drought stress (Tong et al., 2020). In the present study, the genes (i.e., *ANR, E2.3.1.133, CHS, CYP98A, CYP75B1*, and *C3’H*) involved in the biosynthesis of flavonoids were up-regulated under drought stress. The flavonoid contents were also higher in the D2 and D3 treatments than in CK. The results implied that the species regulate the expression of genes related to flavonoid biosynthesis and increase the flavonoid content to response to drought stress. Tong et al. (2020) also found the accumulation of flavonoids, amino acid, and organic acids in moso bamboo (*Phyllostachys edulis*) under drought stress and characterized its response to drought stress in the field. MDA was generated by the reaction of polyunsaturated fatty acids and oxygen species and could be used to evaluate oxidative damage (Rathore et al., 2018). The results showed that MDA content was significantly different under drought stress, suggesting that the drought treatments resulted in oxidative damage or excessive oxidation in *R. rex*. These results suggested that the related physiological process to drought resistance was regulated by genes involved in sugar metabolism and biosynthesis of flavonoids.

The cell wall is essential for the perception and response to adverse environments to regulate the growth and development in plants (Singh et al., 2017). Fox et al. (2018) demonstrated that the cell wall-related transcripts (expansin) were enriched when *Pinus halepensis* was under drought stress and water recovery treatment. This result indicated that the related cell wall metabolism may play a vital role in the response to drought stress. The present analysis of the DEGs showed that the cell wall macromolecule catabolic and metabolic processes were significantly enriched, and the related genes (i.e., *CHIB, E2.4.1.207*, and *E3.2.1.14*) were highly expressed under drought stress. Conversely, the genes (i.e., *GALS, E3.1.1.11, HHT1, pel,* and *GAUT*) involved in pectin and galacturonan metabolism were lowly expressed under drought treatments. The genes of *CHIB* were not only involved in the cell wall macromolecule catabolic and metabolic processes but also in the MAPK signaling pathway. In addition, *CHIB* was involved in the amino sugar and nucleotide sugar metabolism. In the MAPK signaling pathway, CHIB was activated and then directed defense responses. Similar to *CHIB, E3.2.1.14* was not only involved in the cell wall macromolecule catabolic and metabolic processes but also in the amino sugar and nucleotide sugar metabolism. *E3.2.1.14* is a key gene in UDP sugar metabolism. Pectin is mainly composed of the homopolymers of galacturonic acid and maintains proper cell wall structure and function by promoting the binding of Ca^2+^ with the esterified carboxyl groups of galacturonic acid (Maejima et al., 2014). Pel is a pectate lyase and can degrade deesterified pectin to loosen the cell wall, resulting in remodeling and rearrangement (Sun et al., 2018). Furthermore, pel participates in plant growth and development by regulating the auxin signal pathway (Sun et al., 2018). GAUT is a galacturonosyltransferase that directly participates in pectin synthesis, and the pectin and xylan were affected by GAUT as shown in a study of *Arabidopsis thaliana* (Caffall et al., 2009). Therefore, the down-regulation of these genes would lead to the inhibition of pectin and galacturonan metabolism or biosynthesis under drought stress. The results indicated that the cell wall structures and functions were likely altered by the regulation of genes involved in pectin and galacturonan metabolic processes or biosynthesis during the response of *R. rex* to drought stress.

## Conclusion

Although numerous studies have been conducted on the response or tolerance to drought stress of crops and model plants, investigations on the mechanism of such response by alpine woody plant are severely deficient. This deficiency will affect the understanding of the response of forest ecosystems to the undergoing global climate change. In the present study, we first identified the transcriptomic, physiological, and biochemical indicators of the response of alpine woody plant *R. rex* to drought stress by using gene co-expression network and regression analysis. The findings showed that the molecular pathways involved in photosynthesis, sugar metabolism, biosynthesis of flavonoids, and phytohormone signal pathways would play crucial roles in the response of *R. rex* to drought stress. In addition, genes associated with cell wall, pectin, and galacturonan metabolism that also played crucial roles in such response of the studied species. The physiological and biochemical indicators, including Y(II), ETR, qP, Fv/Fm, chlorophyll content, TSS, proline, and MDA, also participated in the response of *R. rex* to drought stress. The present study provides new insights into the understanding of the response mechanism of alpine woody plants to drought stress and may enhance the knowledge of the response of forest ecosystems to global climate change.

## Data Availability Statement

The datasets presented in this study can be found in online repositories. The names of the repository/repositories and accession number(s) can be found at: National Center for Biotechnology Information (NCBI) BioProject database under accession number PRJNA738298.

## Author Contributions

S-KS, X-LZ, and Y-HW conceived and designed the experiment. X-LZ, J-YM, Z-DL, N-fD, H-QY, and LY performed the experiments. X-LZ, J-YM, Z-DL, N-fD, and LY analyzed the data. S-KS, X-LZ, and Y-HW wrote and approved the manuscript. All authors contributed to the article and approved the submitted version.

## Funding

This study was financially supported by the National Natural Science Foundation of China (31870529 and 31560224), the Major Program for Basic Research Project of Yunnan Province (202101BC070002), the Young Academic and Technical Leader Raising Foundation of Yunnan Province (2018HB035), the Open Fund of Yunnan Key Laboratory for Plateau Mountain Ecology and Restoration of Degraded Environments (2018DG005), and the Program for Excellent Young Talents.

## Conflict of Interest

The authors declare that the research was conducted in the absence of any commercial or financial relationships that could be construed as a potential conflict of interest.

## Publisher’s Note

All claims expressed in this article are solely those of the authors and do not necessarily represent those of their affiliated organizations, or those of the publisher, the editors and the reviewers. Any product that may be evaluated in this article, or claim that may be made by its manufacturer, is not guaranteed or endorsed by the publisher.

## Footnotes

^1^ https://github.com/jstjohn/SeqPrep

^2^ https://github.com/trinityrnaseq/trinityrnaseq

^3^ www.majorbio.com
