# Supplementary material for: Gene Co-expression Network and Regression Analysis Identify the Transcriptomic, Physiological, and Biochemical Indicators of the Response of Alpine Woody Plant Rhododendron rex to Drought Stress
Source: Front Plant Sci. 2022 May 25;13:896691. doi: 10.3389/fpls.2022.896691 (PMC9174646; doi:10.3389/fpls.2022.896691)
Supplement: Supplementary file 1 [file Data_Sheet_1.PDF]

Table S1 Primers for Real Time PCR in this study

Genes	Primers	5’----3’	Length(bp)
PYL	PYL_F	5' TAATGGATTTAGCAGCGT 3'	286
	PYL_R	5' CTCCTCGTCATCAAGAAG 3'	
psbS	psbS_F	5' GCCTACTTTTTCTCTTTC 3'	290
	psbS_R	5' ATATTTAATCTTCCGCTT 3'	
HSP90A	HSP90A_F	5' GACGACAGAAAAGGAAAT 3'	222
	HSP90A_R	5' CTCTTGTAGAAAGCAGCA 3'	
SOD1	SOD1_F	5' GATCCATCAAACACAGAG 3'	152
	SOD1_R	5' AATCAAGTTACAACCCAA 3'	
ARF	ARF-F	5' AAATGGCATATACAAGGGAC 3'	260
	ARF-R	5' TTTGGAAACACAACTCAGGA 3'	
PPP2C	PPP2C-F	5' AGCAATTCAACCATACCAAC 3'	148
	PPP2C-R	5' ATAGAAGCCATATTTCCACA 3'	
SNRK2	SNRK2-F	5' GCTTTCAAACGCATCCCTTA 3'	232
	SNRK2-R	5' CCCTTGCCACATTCTCATCA 3'	
CYP	F	5' AGGGCCGAAATGTCGTTCTA3'	164
	R	5' CCCATCTCCAGCCAAATCAT3'	

Table S2 Summary statistics of *de novo* assembled transcriptome for *Rhododendron rex*.

Category	Items	Number
Raw data	Total raw reads	49690398
Clean data	Total clean reads	49340495.13
	Error%	0.025
	Q20%	98.10
	Q30%	94.05
	GC%	46.75
Transcripts	Total number	255300
	Smallest length(bp)	201
	Largest length(bp)	15780
	Mean length(bp)	1034.85
	N50(bp)	1754
Unigenes	Total number	148136
	Smallest length(bp)	201
	Largest length(bp)	15780
	Mean length(bp)	918.42
	N50(bp)	1525

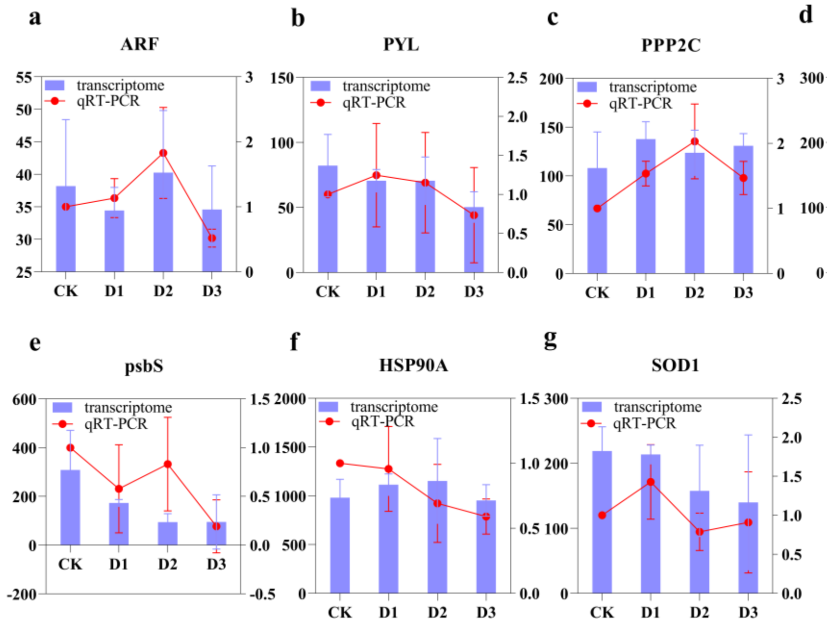

Figure S1 The kinetics of gene expression changes in seedlings of *Rhododendron rex* after under drought stress. Transcript abundance of seven genes was determined by real-time RT-PCR. The broken line represents the values of the qRT-PCR, and the column diagram represents the values of the transcriptome. The figure shows the average relative mRNA levels ± SEM for three biological replicates.

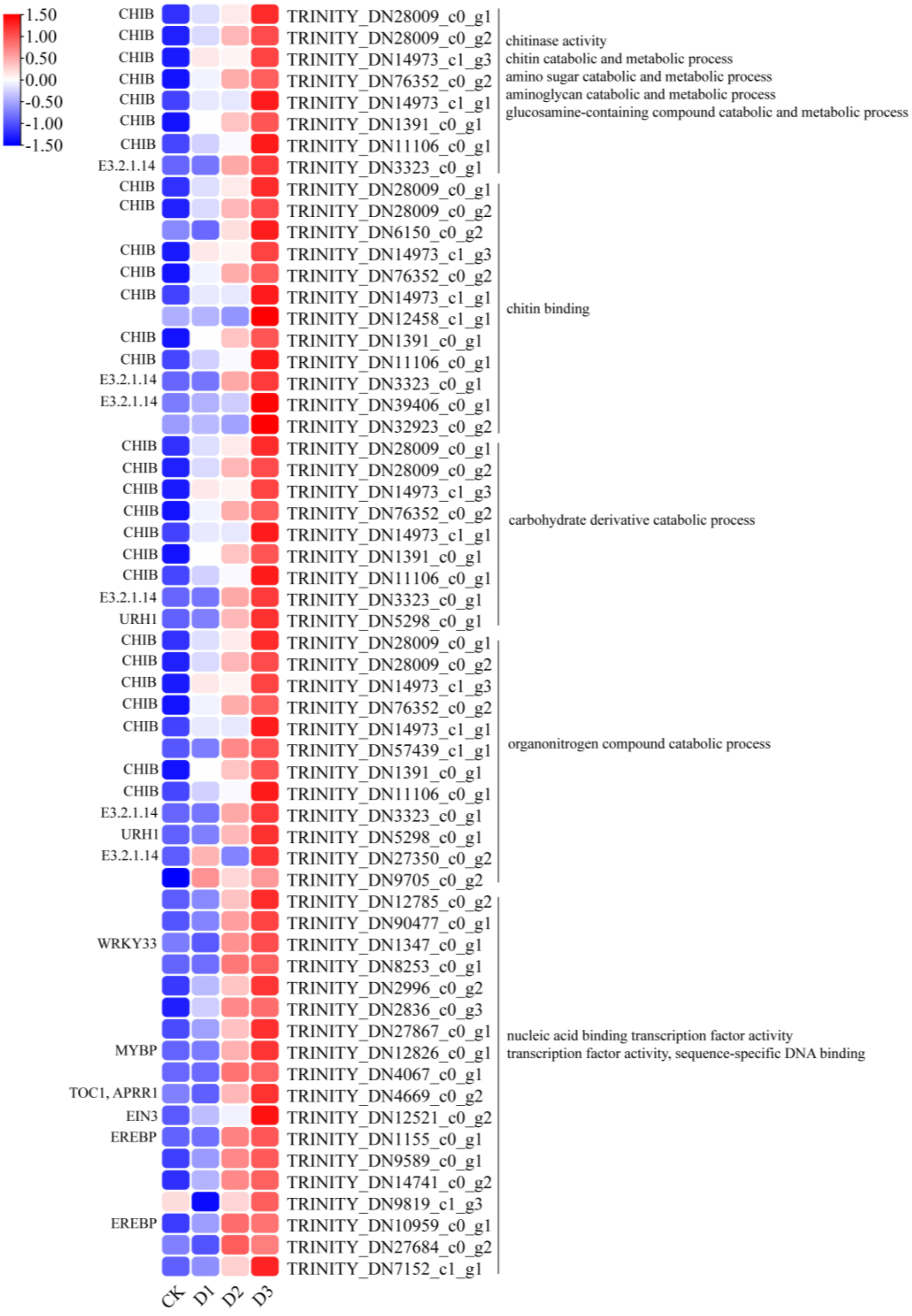

Figure S2 The up-regulated unigenes was significant enrichened of GO items in the CK verse D3. The color was showed the expression values (log_{10}(TPM)), and the red represent high TPM. The figures were visualized by TBtools.

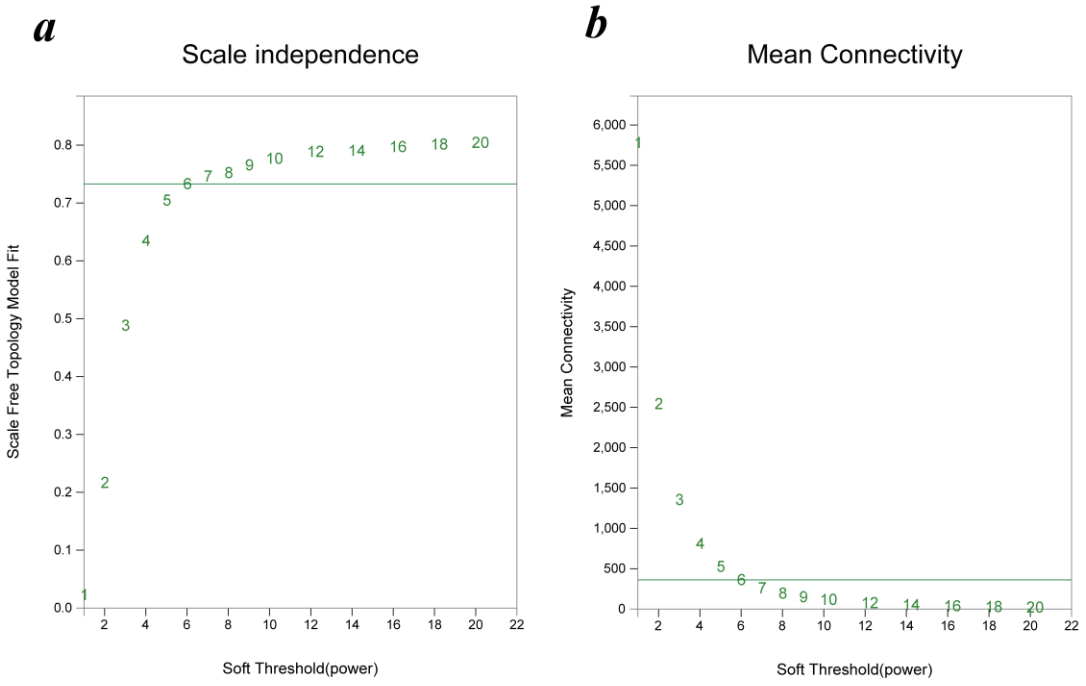

Figure S3 The co-expression network analysis. (a) Selection of the soft-thresholding powers. The scale-free fit index vs. soft-thresholding power. (b) The mean connectivity vs. soft-thresholding power.

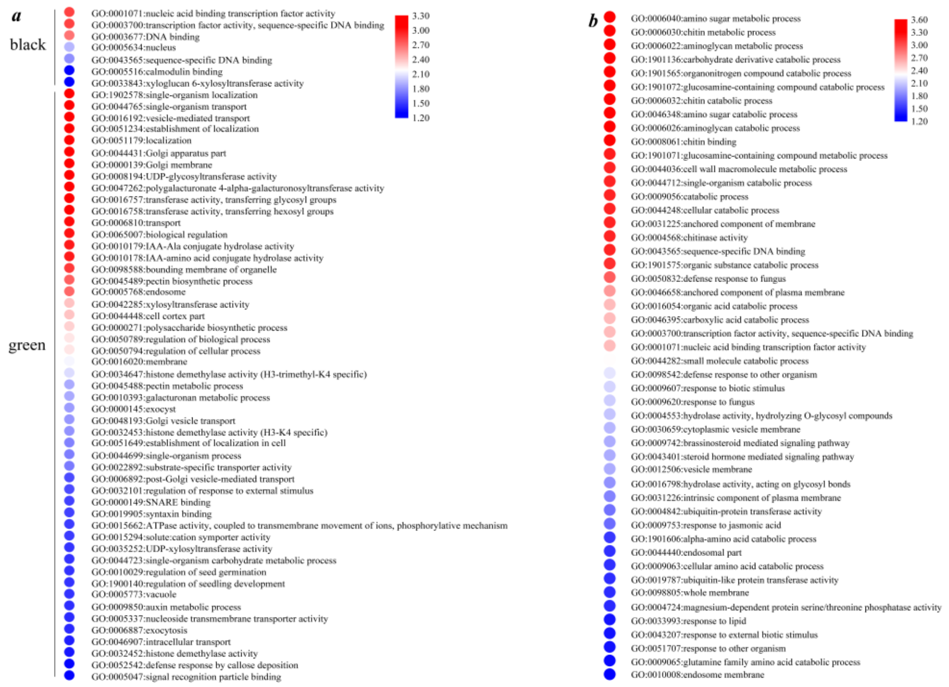

Figure S4 The module unigenes was significant enrichened (P value corrected < 0.05) of GO. a: balck and green modules; b: red module. The color was represented -log (P value corrected), the red was showed the low P value corrected and the blue was high P value corrected. The figures were visualized by TBtools.

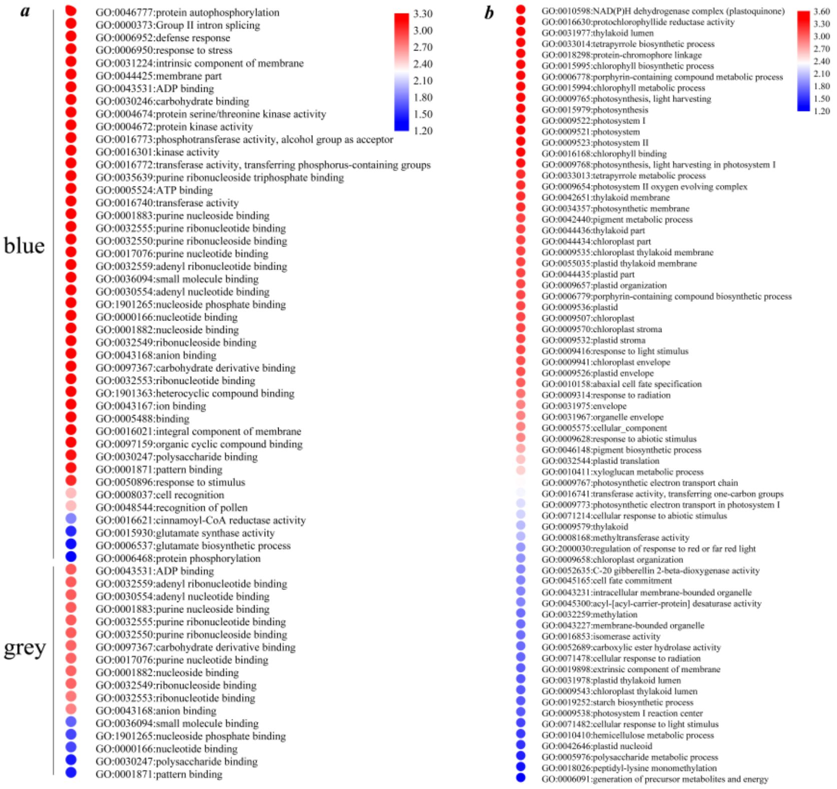

Figure S5 The module unigenes was significant enrichened (P value corrected < 0.05) of GO. a: blue and grey modules; b: brown module. The color was represented -log (P value corrected), the red was showed the low P value corrected and the blue was high P value corrected. The figures were visualized by TBtools.
